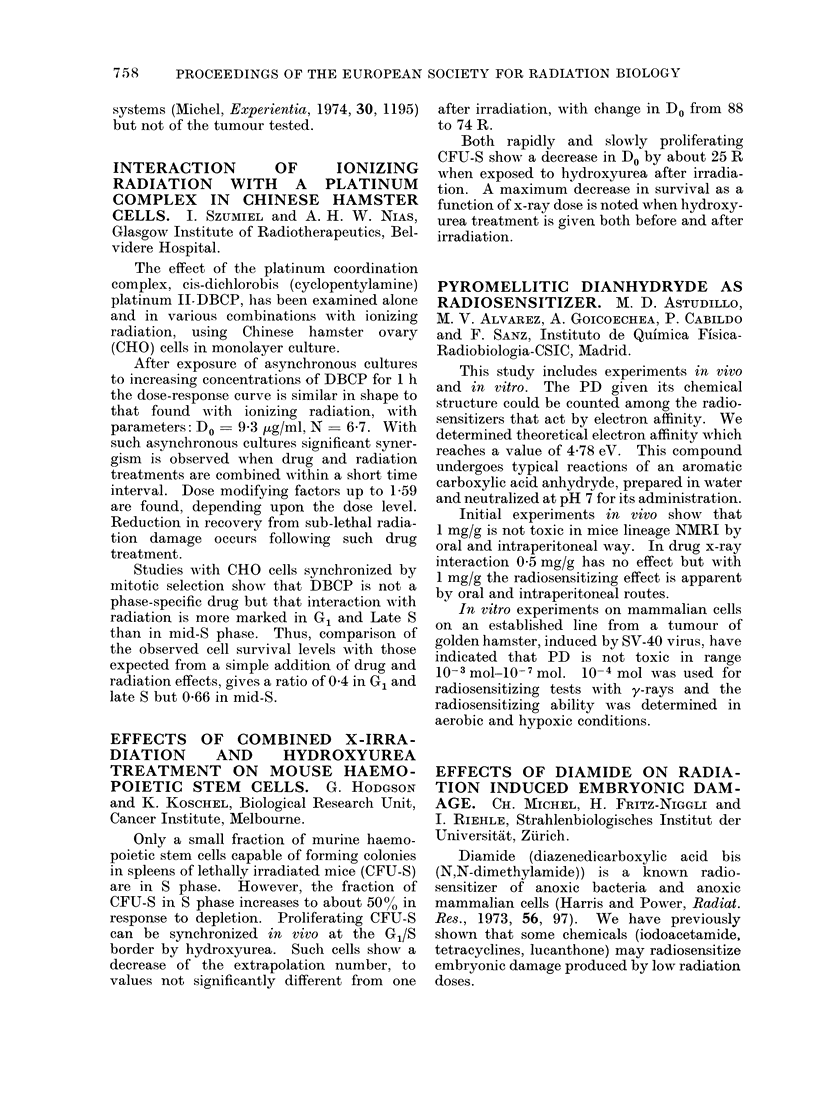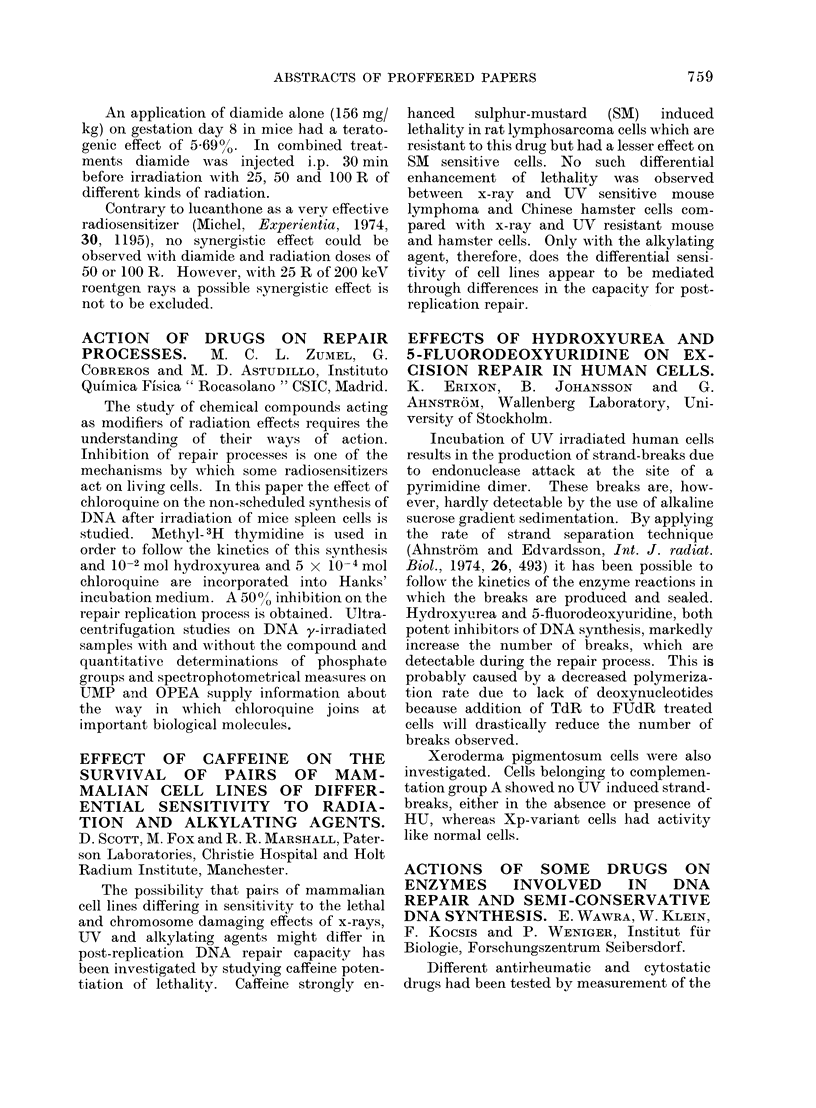# Proceedings: Effects of diamide on radiation induced embryonic damage.

**DOI:** 10.1038/bjc.1975.314

**Published:** 1975-12

**Authors:** C. Michel, H. Fritz-Niggli, I. Riehle


**EFFECTS OF DIAMIDE ON RADIATION INDUCED EMBRYONIC DAMAGE.** Ch. Michel, H. Fritz-Niggli and I. Riehle, Strahlenbiologisches Institut der Universität, Zürich.

Diamide (diazenedicarboxylic acid bis (N,N-dimethylamide)) is a known radiosensitizer of anoxic bacteria and anoxic mammalian cells (Harris and Power, *Radiat. Res.*, 1973, **56**, 97). We have previously shown that some chemicals (iodoacetamide, tetracyclines, lucanthone) may radiosensitize embryonic damage produced by low radiation doses.

An application of diamide alone (156 mg/kg) on gestation day 8 in mice had a teratogenic effect of 5·69%. In combined treatments diamide was injected i.p. 30 min before irradiation with 25, 50 and 100 R of different kinds of radiation.

Contrary to lucanthone as a very effective radiosensitizer (Michel, *Experientia*, 1974, **30**, 1195), no synergistic effect could be observed with diamide and radiation doses of 50 or 100 R. However, with 25 R of 200 keV roentgen rays a possible synergistic effect is not to be excluded.